# 外周血炎症指标作为预测性指标在晚期非小细胞肺癌免疫治疗中的应用

**DOI:** 10.3779/j.issn.1009-3419.2021.103.10

**Published:** 2021-09-20

**Authors:** 经纬 夏, 羽中 陈, 少迪 温, 晓月 杜, 波 沈

**Affiliations:** 210009 南京，南京医科大学附属肿瘤医院/江苏省肿瘤医院/江苏省肿瘤防治研究所 The Affiliated Cancer Hospital of Nanjing Medical University, Jiangsu Cancer Hospital, Jiangsu Institute of Cancer Research, Nanjing 210009, China

**Keywords:** 肺肿瘤, 免疫检查点抑制剂, 中性粒细胞与淋巴细胞计数比值, 列线图, Lung neoolasms, Immune checkpoint inhibitors, Neutrophil-to-lymphocyte ratio, Nomogram

## Abstract

**背景与目的:**

肺癌是癌症相关死亡最主要的病因，其中非小细胞肺癌（non small cell lung cancer, NSCLC）是最常见的类型。目前免疫检查点抑制剂（immune checkpoint inhibitors, ICIs）已经成为晚期NSCLC主要的治疗方法之一。本文回顾性研究了外周血炎症指标对晚期NSCLC患者免疫治疗疗效及生存预后的影响，以寻找指导NSCLC免疫治疗的策略。

**方法:**

选取2018年10月-2019年8月于南京医科大学附属肿瘤医院住院治疗的晚期NSCLC患者，均接受抗PD-1（Pembrolizumab、Sintilimab或Toripalimab）单药或者联合方案治疗。随访至2020年12月10日，根据RECIST1.1标准评价疗效，分析影响疗效的显著变量，并随访无进展生存期（progression-free survival, PFS）及总生存期（overall survival, OS）进行生存分析。根据治疗前、治疗后6周、治疗后12周（0 w、6 w、12 w）三个不同时间点中性粒细胞计数与淋巴细胞计数比值（neutrophil-to-lymphocyte ratio, NLR）数据构建临床预测模型分析NLR的预测价值，并验证模型准确性。

**结果:**

最终纳入173例患者，所有患者均接受上述治疗方案，中位随访时间19.7个月。客观缓解率（objective response rate, ORR）27.7%（48/173），疾病控制率（disease control rate, DCR）89.6%（155/173），中位PFS为8.3个月（7.491-9.109），中位OS为15.5个月（14.087-16.913）。*χ*^2^检验及*Logistic*多因素分析显示NLR_6w_与ORR相关，NLR_12w_与ORR、DCR相关，进一步*Cox*回归分析显示NLR_6w_和NLR_12w_影响PFS，NLR_0w_、NLR_6w_和NLR_12w_与OS相关。

**结论:**

在晚期NSCLC患者中，不同时间点的NLR数值是免疫治疗反应的有效预测因子，并且NLR < 3往往与良好的预后相关。

## Abstract (en)

**Background and objective:**

Lung cancer is the leading cause of cancer-related death, of which non-small cell lung cancer (NSCLC) is the most common type. Immune checkpoint inhibitors (ICIs) have now become one of the main treatments for advanced NSCLC. This paper retrospectively investigated the effect of peripheral blood inflammatory indexes on the efficacy of immunotherapy and survival of patients with advanced non-small cell lung cancer, in order to find strategies to guide immunotherapy in NSCLC.

**Methods:**

Patients with advanced non-small cell lung cancer who were hospitalized in The Affiliated Cancer Hospital of Nanjing Medical University from October 2018 to August 2019 were selected to receive anti-PD-1 (pembrolizumab, sintilimab or toripalimab) monotherapy or combination regimens. And were followed up until 10 December 2020, and the efficacy was evaluated according to RECIST1.1 criteria. Progression-free survival (PFS) and overall survival (OS) were followed up for survival analysis. A clinical prediction model was constructed to analyze the predictive value of neutrophil-to-lymphocyte ratio (NLR) based on NLR data at three different time points: before treatment, 6 weeks after treatment and 12 weeks after treatment (0w, 6w and 12w), and the accuracy of the model was verified.

**Results:**

173 patients were finally included, all of whom received the above treatment regimen, were followed up for a median of 19.7 months. The objective response rate (ORR) was 27.7% (48/173), the disease control rate (DCR) was 89.6% (155/173), the median PFS was 8.3 months (7.491-9.109) and the median OS was 15.5 months (14.087-16.913). The *chi-square* test and *logistic* multi-factor analysis showed that NLR_6w_ was associated with ORR and NLR_12w_ was associated with ORR and DCR. Further *Cox* regression analysis showed that NLR_6w_ and NLR_12w_ affected PFS and NLR_0w_, NLR_6w_ and NLR_12w_ were associated with OS.

**Conclusion:**

In patients with advanced non-small cell lung cancer, NLR values at different time points are valid predictors of response to immunotherapy, and NLR < 3 is often associated with a good prognosis.

根据国际癌症研究机构编制的GLOBOCAN的2020年最新数据统计，肺癌仍是全球癌症相关死亡的首位病因，每年有18%的肺癌患者死亡，而非小细胞肺癌（non-small cell lung cancer, NSCLC）发病率约占所有肺癌患者的80%-85%，且大多数患者诊断时已是晚期，晚期患者的5年生存率更是仅为19%^[1]^。目前以程序性死亡受体1（programmed cell death 1, PD-1）/程序性死亡配体1（programmed cell death-ligand 1, PD-L1）免疫检查点抑制剂（immune checkpoint inhibitors, ICIs）为代表的免疫治疗对NSCLC患者具有显著的疗效。KEYNOTE-001研究^[2]^报道的一线接受Pembrolizumab治疗的晚期NSCLC患者5年总生存率为23.2%。ICIs单药或联合治疗已成为驱动基因阴性晚期NSCLC患者的一线标准治疗。然而免疫治疗可能带来严重的免疫相关不良反应，因此，准确有效的生物标志物对于确定免疫治疗是否能够获益十分关键。目前，PD-L1和肿瘤突变负荷（tumor mutation burden, TMB）仍然是预测NSCLC免疫治疗疗效的最常见的生物标志物，还有许多潜在的预测性生物标志物，包括微生物组、肿瘤浸润淋巴细胞、基因标志、多组学等^[3]^。但是这类生物标志物检测方法较为复杂、费用昂贵、耗时久，这限制了它们的临床应用。因此需要可以简易获取的生物标志物来选择免疫治疗的潜在受益人群。

近年来，炎症反应在肿瘤微环境中的重要作用逐渐被发现，肿瘤微环境中炎症因子在肿瘤的发生、发展、侵袭和转移中起重要作用，如白细胞介素-6（interleukin-6, IL-6），肿瘤坏死因子-α（tumor necrosis factor-α, TNF-α）和转化生长因子-β（transforming growth factor-β, TGF-β）等^[4]^。在诸多炎症指标中，中性粒细胞与淋巴细胞比值（neutrophil-to-lymphocyte ratio, NLR）、血小板与淋巴细胞比值（platelet-to-lymphocyte ratio, PLR）被证实与恶性肿瘤有明显的相关性，可为预后提供重要的信息^[5-9]^。然而基线值以及治疗后不同时期数值的临床意义仍有争议，因此，我们进行了一项回顾性研究，分析外周血炎症标志物对晚期NSCLC患者免疫治疗疗效和生存预后的预测价值。具体包括：中性粒细胞计数（absolute neutrophil counts, ANC）、淋巴细胞计数（absolute lymphocyte counts, ALC）、血小板计数（platelet count, PLT）、中性粒细胞与淋巴细胞比值（neutrophil-to-lymphocyte ratio, NLR）及血小板与淋巴细胞比值（platelet-lymphocyte ratio, PLR）。

## 1 资料与方法

### 1.1 临床资料

回顾性分析2018年10月-2019年8月于中国南京医科大学附属肿瘤医院接受抗PD-1治疗（Pembrolizumab、Sintilimab或Toripalimab）的173例晚期NSCLC患者。所有患者均符合以下标准：入组标准：①病理学明确诊断为NSCLC；②初诊时临床分期为不可手术Ⅲ期或Ⅳ期；③接受过抗PD-1免疫治疗（单药或联合方案）；④病历资料完整，存在可评估疗效的影像学资料。排除标准：①1个月内出现炎症性疾病或接受手术；②近期使用过类固醇药物；③治疗期间发生严重副反应，如骨髓抑制、肝功能受损等；④出现重度贫血、营养不良。

最终纳入符合标准患者173例，其中抗PD-1单药方案44例，联合化疗方案83例，联合抗血管生成药物方案18例，联合化疗和抗血管生成药物方案28例。

通过电子病历或电话随访收集患者的临床病理特征，包括治疗时的年龄、性别、病理类型、肿瘤原发灶-淋巴结-转移（tumor-node-metastasis, TNM）分期^[10]^、东部合作肿瘤小组评分（Eastern Cooperative Oncology Group, ECOG）、驱动基因突变类型、吸烟史、治疗线数等。分别于治疗前、治疗后第6周和第12周（0 w、6 w、12 w）收集患者的血液学指标，包括ANC、ALC、PLT、NLR以及PLR。本研究由江苏省肿瘤医院机构评审委员会批准。

### 1.2 研究方法

#### 1.2.1 治疗方案

具体治疗方案如下：单药方案患者每3周接受一次Pembrolizumab静脉注射固定剂量200 mg，Sintilimab静脉注射固定剂量200 mg，或Toripalimab静脉注射固定剂量240 mg。联合化疗为含铂双药化疗，其他药物根据肿瘤组织学确定，包括培美曲塞、多西紫杉醇、紫杉醇/白蛋白紫杉醇、吉西他滨。联合抗血管生成药物为贝伐珠单抗。其中存在一部分具有*EGFR*突变患者，其PD-1抑制剂免疫治疗的使用均为晚期后线治疗。

#### 1.2.2 疗效评价及随访

通过电子病历以及电话随访获得数据，随访起止时间为2018年10月1日-2020年12月10日。治疗后每6周-8周进行一次全身电子计算机断层扫描（computed tomography, CT），根据实体瘤的评价标准（Response Evaluation Criteria in Solid Tumour 1.1, RECIST1.1）标准评估患者对治疗的疗效，分为完全缓解（complete response, CR）、部分缓解（partial response, PR）、疾病稳定（stable disease, SD）和疾病进展（progressive disease, PD）。以客观缓解率（objective response rate, ORR）和疾病控制率（disease control rate, DCR）评价疗效，以无进展生存期（progression-free survival, PFS）和总生存期（overall survival, OS）评价生存率。ORR定义为CR和PR之和，DCR定义为CR、PR和SD之和。PFS定义为从初始治疗到临床或影像学进展或死亡的时间，OS定义为从初始治疗到最后一次随访或死亡的时间。

### 1.3 统计学方法

采用SPSS 25.0、GraphPad Prism 8.3.0以及R等统计学软件进行数据分析与绘图。根据本研究数据中位数结合既往研究及相关文献，NLR截断值取3，PLR截断值取160^[11-13]^。使用*χ*^2^检验及*Logistic*回归多因素分析确定影响DCR及ORR的显著变量。采用*Kaplan-Meier*法进行生存分析，绘制OS及PFS曲线，并采用*Log-rank*检验对生存曲线进行组间比较。采用*Cox*比例风险模型确定PFS、OS的预后因素。基于*Cox*多因素分析结果，构建列线图，并使用自举重采样（bootstrap resampling）进行验证，利用一致性指数（c-index）以及校准曲线评估预测模型的准确性。运用单因素分析对预后因素初筛，变量筛选的检验水准设为α=0.10，即把单因素分析*P* < 0.10的因素纳入多因素回归分析。*P* < 0.05认为有统计学差异。

## 2 结果

### 2.1 基线资料

共173例患者纳入研究，6例患者2个周期治疗后因副反应明显、死亡或其他不明原因未能继续后续治疗，其余167例患者均完成4个周期及以上的治疗并有可评估疗效的影像学资料。中位年龄为64岁；男性134例（77.5%），女性39例；65.9%的患者为非鳞癌；79.8%的患者为Ⅳ期患者；97.7%患者ECOG评分为0分-1分，只有4例患者ECOG评分为2分；33例患者携带*EGFR*基因突变；62.4%的患者有吸烟史；初治患者67例（38.7%），复治患者106例（61.3%）；其中接受过放疗的患者占45.7%；使用Pembrolizumab、Sintilimab和Toripalimab的患者数分别为75例（43.4%）、79例（45.7%）和19例（11.0%）；25.4%（44/173）的患者接受单药方案，几乎一半患者（48.0%）接受ICIs联合化疗方案治疗，10.4%（18/173）患者接受ICIs联合抗血管生成药物方案，16.2%（28/173）患者接受ICIs联合化疗、抗血管生成药物方案治疗（表 1）。患者各阶段实验室检查结果详见表 2。

**表 1:** 173例患者的临床特征 Clinical characteristics of 173 patients

Characteristics		*n*	Percent (%)
Patients		173	
Age (yr)	≥64	85	49.1
	< 64	88	50.9
Gender	Male	134	77.5
	Female	39	22.5
Histology	Squamous carcinoma	59	34.1
	Non-squamous carcinoma	114	65.9
TNM stage	Ⅲ	35	20.2
	Ⅳ	138	79.8
ECOG PS	0-1	169	97.7
	2	4	2.3
*EGFR* mutation	Yes	33	19.1
	No	140	80.9
Smoking	Yes	108	62.4
	No	65	37.6
Lines of treatment	1	67	38.7
	≥2	106	61.3
Radiotherapy	Yes	79	45.7
	No	94	54.3
ICIs	Pembrolizumab	75	43.4
	Sintilimab	79	45.7
	Toripalimab	19	11.0
Option of treatment	ICIs	44	25.4
	ICIs+Chemotherapy	83	48.0
	ICIs+Anti-angiogenic	18	10.4
	ICIs+Chemotherapy+Anti-angiogenic	28	16.2
ECOG: Eastern Cooperative Oncology Group; EGFR: epidermal growth factor receptor; ICIs: immune checkpoint inhibitors; TNM: tumor-node-metastasis.			

**表 2:** 173例患者的实验室检查结果、治疗反应及生存 Laboratory tests and scores, response to treatment and survival of 173 patients

Laboratory tests and scores		Data	Percent (%)
Before treatment			
ANC (×10^9^/L) [Median (IQR)]		4.130 (3.135-5.440)	
ALC (×10^9^/L) [Median (IQR)]		1.450 (1.045-1.795)	
PLT (×10^9^/L) [Median (IQR)]		221.0 (174.5-271.0)	
NLR	≥3	78	45.1
	< 3	95	54.9
PLR	≥160	83	48.0
	< 160	90	52.0
6 weeks after treatment			
ANC (×10^9^/L) [Median (IQR)]		4.040 (2.905-5.635)	
ALC (×10^9^/L) [Median (IQR)]		1.340 (0.985-1.840)	
PLT (×10^9^/L) [Median (IQR)]		217.0 (173.0-281.5)	
NLR	≥3	81	46.8
	< 3	92	53.2
PLR	≥160	84	48.6
	< 160	89	51.4
12 weeks after treatment			
ANC (×10^9^/L) [Median (IQR)]		3.890 (2.940-5.720)	
ALC (×10^9^/L) [Median (IQR)]		1.320 (0.920-1.780)	
PLT (×10^9^/L) [Median (IQR)]		206.0 (159.0-257.0)	
NLR	≥3	77	46.1
	< 3	90	53.9
PLR	≥160	72	43.1
	< 160	95	56.9
Response and survival			
Best overall response	CR	0	0
	PR	48	27.7
	SD	107	61.8
	PD	18	10.4
	NE	0	0
Response rate	ORR (%)	48	27.7
	DCR (%)	155	89.6
Survival time	mPFS (mon), 95%CI	8.3	7.491-9.109
	mOS (mon), 95%CI	15.5	14.087-16.913
ANC: absolute neutrophil counts; ALC: absolute lymphocyte counts; PLT: platelet count; NLR: neutrophil-to-lymphocyte ratio; PLR: platelet-to-lymphocyte ratio; IQR: interquartile range; CR: complete response; PR: partial response; SD: stable disease; PD: progressive disease; NE: not evaluated; ORR: objective response rate; DCR: disease control rate; PFS: progression-free survival; OS: overall survival.			

### 2.2 疗效评价

随访时间截至2020年12月10日，全组患者中位随访时间为19.70（14.88-24.52）个月。如表 2所示，173例患者中，无人达到CR，48例达到PR，107例达到SD，18例PD。ORR和DCR分别为27.7%（48/173）和89.6%（155/173）。

如表 3所示，通过*χ*^2^检验分析影响疗效的因素，结果显示年龄、吸烟、治疗线数、NLR_12w_影响DCR，年龄、NLR_0w_、NLR_6w_、NLR_12w_与ORR相关。随后的多因素*Logistic*回归分析显示，年龄≥64（OR=4.141, 95%CI: 1.304-13.146）和NLR_12w_ < 3（OR=6.567, 95%CI: 1.392-30.975）与更高的DCR相关；年龄≥64（OR=2.175, 95%CI: 1.093-4.330），NLR_6w_ < 3（OR=2.827, 95%CI: 1.369-5.838）和NLR_12w_ < 3（OR=2.393, 95%CI: 1.178-4.860）与更高的ORR显著相关。

**表 3:** 非小细胞肺癌患者疗效与临床因素的相关分析 Associations between response to treatment and clinical factors in patients with non-small cell lung cancer

Item	Disease control rate			Overall response rate	
	*n* (%)	*P*		*n* (%)	*P*
Age (yr)		0.010			0.025
≥64	71/85 (83.5)			17/85 (20.0)	
< 64	84/88 (95.5)			31/88 (35.2)	
Gender		0.247			0.632
Male	122/134 (91.0)			36/134 (26.9)	
Female	33/39 (84.6)			12/39 (30.8)	
Histology		0.550			0.624
Squamous carcinoma	54/59 (91.5)			15/59 (25.4)	
Non-squamous carcinoma	101/114 (88.6)			33/114 (28.9)	
TNM stage		0.309			0.333
Ⅲ	33/35 (94.3)			12/35 (34.3)	
Ⅳ	122/138 (88.4)			36/138 (26.1)	
ECOG PS		0.333			0.210
0-1	152/169 (89.9)			48/169 (28.4)	
2	3/4 (75.0)			0/4 (0.0)	
*EGFR* mutation		0.104			0.173
Yes	27/33 (81.8)			6/33 (18.2)	
No	128/140 (91.4)			42/140 (30.0)	
Smoking		0.096			0.164
No	55/65 (84.6)			22/65 (33.8)	
Yes	100/108 (92.6)			26/108 (24.1)	
Lines of treatment		0.042			0.401
1	64/67 (95.5)				
≥2	91/106 (85.8)				
Radiotherapy		0.108			0.978
Yes	74/79 (93.7)				
No	81/94 (86.1)				
Option of treatment		0.809			0.638
Monotherapy	39/44 (88.6)				
Combination therapy	116/129 (89.9)				
Before treatment					
NLR		0.954			0.903
≥3	70/78 (89.7)				
< 3	85/95 (89.5)				
PLR		0.856			0.727
≥160	74/83 (89.2)				
< 160	81/90 (90.0)				
6 weeks after treatment					
NLR		0.199			0.004
≥3	70/81 (86.4)				
< 3	85/92 (92.4)				
PLR		0.104			0.143
≥160	72/84 (85.7)				
< 160	83/89 (93.3)				
12 weeks after treatment					
NLR		0.007			0.014
≥3	67/77 (87.0)				
< 3	88/90 (97.8)				
PLR		0.269			0.202
≥160	65/72 (90.3)				
< 160	90/95 (94.7)				

### 2.3 生存分析

总人群的中位PFS及OS分别为8.3个月（95%CI: 7.491-9.109）和15.5个月（95%CI: 14.087-16.913）。对PFS及OS分别进行单因素与多因素*Cox*回归分析，发现ECOG评分0-1组患者PFS较长（HR=0.196, 95%CI: 0.070-0.550, *P*=0.002），接受放疗可以降低患者的疾病进展风险（HR=0.602, 95%CI: 0.413-0.877, *P*=0.008），单药方案也显示出更好的控制疾病进展的能力（HR=0.489, 95%CI: 0.303-0.787, *P*=0.003），NLR_6w_≥3（HR=1.882, 95%CI: 1.296-2.732, *P*=0.001）和NLR_12w_≥3（HR=1.536, 95%CI: 1.048-2.251, *P*=0.028）组患者疾病进展风险较高；*EGFR*突变组（HR=1.956, 95%CI: 1.127-3.395, *P*=0.017），NLR_0w_≥3组（HR=1.700, 95%CI: 1.119-2.584, *P*=0.013），NLR_6w_≥3组（HR=1.719, 95%CI: 1.129-2.616, *P*=0.012）以及NLR_12w_≥3组（HR=2.015, 95%CI: 1.319-3.079, *P*=0.001）患者死亡风险更高，详见表 4、表 5。

**表 4:** 非小细胞肺癌患者PFS单因素与多因素*Cox*回归分析 Univariate and multivariate *Cox* regression analysis of PFS in all patients with non-small cell lung cancer

	Univariate analysis			Multivariate analysis	
	HR (95%CI)	*P*		HR (95%CI)	*P*
Age					
≥64 *vs* < 64	0.822 (0.568-1.190)	0.299		/	/
Gender					
Male *vs* Female	1.032 (0.658-1.618)	0.892		/	/
Histology					
Squamous carcinoma *vs* Non-squamous carcinoma	1.180 (0.807-1.727)	0.393		/	/
TNM stage					
Ⅲ *vs* Ⅳ	1.023 (0.647-1.616)	0.923		/	/
ECOG PS					
0-1 *vs* 2	0.196 (0.070-0.550)	0.002		0.247 (0.088-0.695)	0.008
*EGFR* mutation					
Yes *vs* No	1.252 (0.785-1.997)	0.345		/	/
Smoking					
Yes *vs* No	0.881 (0.604-1.285)	0.509		/	/
Lines of treatment					
1 *vs* ≥2	1.390 (0.948-2.037)	0.092			
Radiotherapy					
Yes *vs* No	0.602 (0.413-0.877)	0.008		0.644 (0.440-0.941)	0.023
Option of treatment					
Monotherapy *vs* Combination therapy	0.489 (0.303-0.787)	0.003		0.522 (0.323-0.844)	0.008
Before treatment					
NLR					
≥3 *vs* < 3	1.265 (0.872-1.835)	0.216		/	/
PLR					
≥160 *vs* < 160	1.265 (0.876-1.827)	0.210		/	/
6 weeks after treatment					
NLR					
≥3 *vs* < 3	1.881 (1.295-2.730)	0.001		1.936 (1.331-2.815)	0.001
PLR					
≥160 *vs* < 160	1.613 (1.116-2.332)	0.011		/	/
ECOG PS					
0-1 *vs* 2	0.196 (0.070-0.550)	0.002		0.238 (0.084-0.673)	0.007
Radiotherapy					
Yes *vs* No	0.602 (0.413-0.877)	0.008		0.619 (0.423-0.905)	0.013
Option of treatment					
Monotherapy *vs* Combination therapy	0.489 (0.303-0.787)	0.003		0.527 (0.325-0.852)	0.009
12 weeks after treatment					
NLR					
≥3 *vs* < 3	1.563 (1.068-2.288)	0.022		1.616 (1.101-2.373)	0.014
PLR					
≥160 *vs* < 160	1.246 (0.853-1.819)	0.256		/	/
ECOG PS					
0-1 *vs* 2	0.196 (0.070-0.550)	0.002		0.230 (0.081-0.656)	0.006
Radiotherapy					
Yes *vs* No	0.602 (0.413-0.877)	0.008		0.617 (0.417-0.912)	0.016
Option of treatment					
Monotherapy *vs* Combination therapy	0.489 (0.303-0.787)	0.003		0.426 (0.252-0.722)	0.002

**表 5:** 非小细胞肺癌患者OS单因素与多因素*Cox*回归分析 Univariate and multivariate *Cox* regression analysis of OS in all patients with non-small cell lung cancer

	Univariate analysis			Multivariate analysis	
	HR (95%CI)	*P*		HR (95%CI)	*P*
Age					
≥64 *vs* < 64	0.749 (0.498-1.126)	0.164		/	/
Gender					
Male *vs* Female	1.085 (0.823-1.430)	0.564		/	/
Histology					
Squamous carcinoma *vs* Non-squamous carcinoma	1.189 (0.773-1.830)	0.431		/	/
TNM stage					
Ⅲ *vs* Ⅳ	1.004 (0.611-1.648)	0.989		/	/
ECOG PS					
0-1 *vs* 2	1.148 (0.281-4.690)	0.848		/	/
*EGFR* mutation					
Yes *vs* No	1.815 (1.052-3.132)	0.032		1.956 (1.127-3.395)	0.017
Smoking					
Yes *vs* No	0.917 (0.594-1.416)	0.697		/	/
Lines of treatment					
1 *vs* ≥2	1.520 (0.993-2.328)	0.054		/	/
Radiotherapy					
Yes *vs* No	1.071 (0.713-1.607)	0.742		/	/
Option of treatment					
Monotherapy vs Combination therapy	0.799 (0.491-1.299)	0.365		/	/
Before treatment					
NLR					
≥3 *vs* < 3	1.622 (1.071-2.457)	0.022		1.700 (1.119-2.584)	0.013
PLR					
≥160 *vs* < 160	1.551 (1.026-2.343)	0.037		/	/
6 weeks after treatment					
NLR					
≥3 *vs* < 3	1.711 (1.125-2.603)	0.012		1.719 (1.129-2.616)	0.012
PLR					
≥160 *vs* < 160	1.040 (0.688-1.571)	0.853		/	/
*EGFR* mutation					
Yes *vs* No	1.815 (1.052-3.132)	0.032		1.828 (1.057-3.158)	0.031
12 weeks after treatment					
NLR					
≥3 *vs* < 3	2.015 (1.319-3.079)	0.001		2.015 (1.319-3.079)	0.001
PLR					
≥160 *vs* < 160	1.114 (0.730-1.700)	0.617		/	/
*EGFR* mutation					
Yes *vs* No	1.815 (1.052-3.132)	0.032		/	/

根据*Cox*回归发现的影响预后的因素分组绘制相应的生存曲线图 1、图 2。发现NLR_6w_ < 3的患者PFS显著长于NLR_6w_≥3的患者（*P* < 0.001），NLR_12w_ < 3组的患者相较于NLR_12w_≥3组也同样有更长的PFS（10.8个月*vs* 7.3个月，*P*=0.02），接受放疗或者单药方案的患者的无进展生存期更长（*P*=0.007, 2, *P*=0.002, 5）。接受ICIs单药治疗的患者多为一线初治患者，这类患者往往有着更加良好的早期疗效。ECOG评分为2的患者无进展生存期较短（4.15个月*vs* 8.5个月，*P* < 0.001），仅有4例患者ECOG评分为2，样本量较少，可能对最终结果有所影响。在OS方面，不论检测时间，NLR < 3组均显示出OS的临床获益。无基因突变患者相较于有*EGFR*突变患者有更长的OS（15.7个月*vs* 15.3个月，*P*=0.035），这一结果也与目前大多数大型研究一致，显示出携带基因突变患者免疫治疗疗效的局限性。

**图 1:**
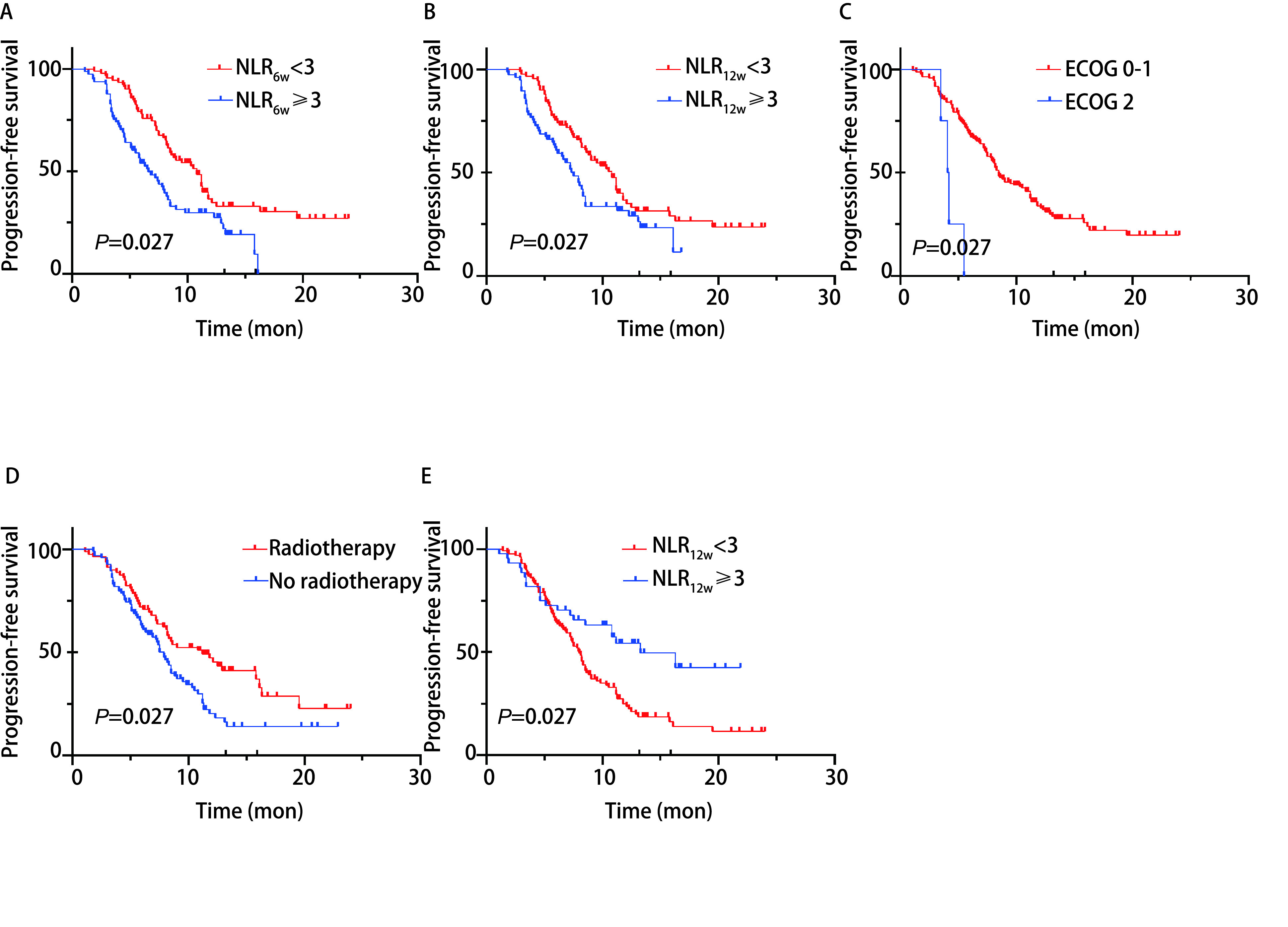
PFS的*Kaplan-Meier*生存分析。A：用药6周后，按NLR分组的患者无进展生存期的*Kaplan-Meier*生存曲线；B：用药12周后，按NLR分组的患者无进展生存期的*Kaplan-Meier*生存曲线；C：按ECOG评分分组的患者无进展生存期的*Kaplan-Meier*生存曲线；D：按患者有无放疗分组的无进展生存期的*Kaplan-Meier*生存曲线；E：不同用药方案的无进展生存期的*Kaplan-Meier*生存曲线。 *Kaplan-Meier* survival analysis for PFS. A: *Kaplan-Meier* survival curves for progression-free survival in patients grouped by NLR after 6 weeks of dosing; B: *Kaplan-Meier* survival curves for progression-free survival in patients grouped by NLR after 12 weeks of dosing; C: *Kaplan-Meier* survival curves for progression-free survival in patients grouped by ECOG PS; D: *Kaplan-Meier* survival curves for progression-free survival in patients grouped by radiotherapy; E: *Kaplan-Meier* survival curves for progression-free survival in patients grouped by option of treatment.

**图 2:**
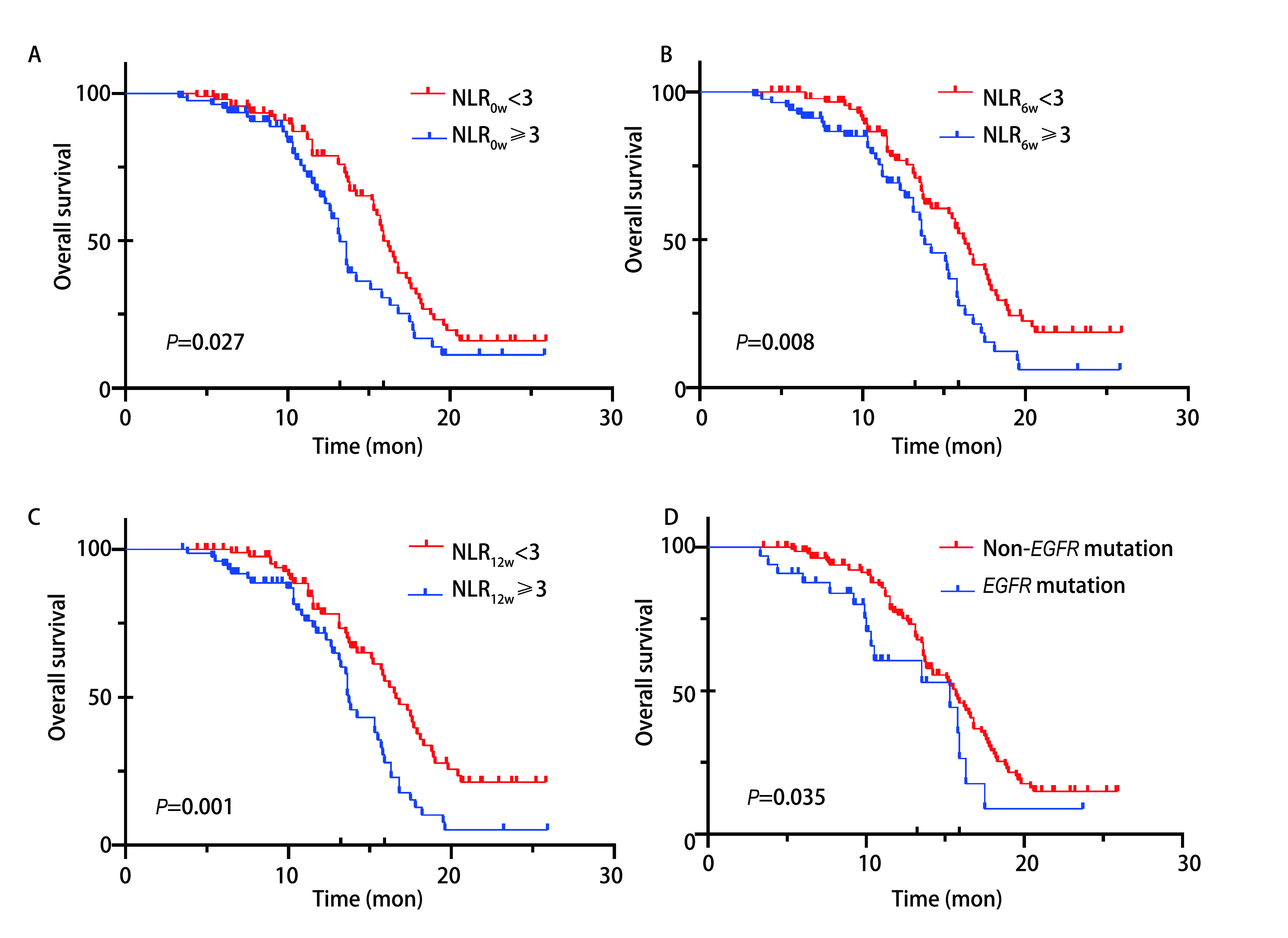
OS的*Kaplan-Meier*生存分析。A：用药前，按NLR分组的患者总生存期的*Kaplan-Meier*生存曲线；B：用药6周后，按NLR分组的患者总生存期的*Kaplan-Meier*生存曲线；C：用药12周后，按NLR分组的患者总生存期的*Kaplan-Meier*生存曲线；D：按是否存在*EGFR*突变分组的患者总生存期的*Kaplan-Meier*生存曲线。 *Kaplan-Meier* survival analysis for OS. A: *Kaplan-Meier* survival curves for overall survival in patients grouped by NLR before dosing; B: *Kaplan-Meier* survival curves for overall survival in patients grouped by NLR after 6 weeks of dosing; C: *Kaplan-Meier* survival curves for overall survival in patients grouped by NLR after 12 weeks of dosing; D: *Kaplan-Meier* survival curves for overall survival in patients grouped by *EGFR* mutation.

### 2.4 列线图评分

基于*Cox*多因素分析结果，构建列线图预测6个月和12个月的无进展生存率及总生存率。NLR_6w_及NLR_12w_分组预测无进展生存率的*c*指数分别为0.640及0.629；NLR_0w_、NLR_6w_及NLR_12w_分组预测总生存率的*c*指数分别为0.603、0.607及0.595。并且各个分组的校准曲线图显示出良好的一致性（图 3、图 4）。

**图 3:**
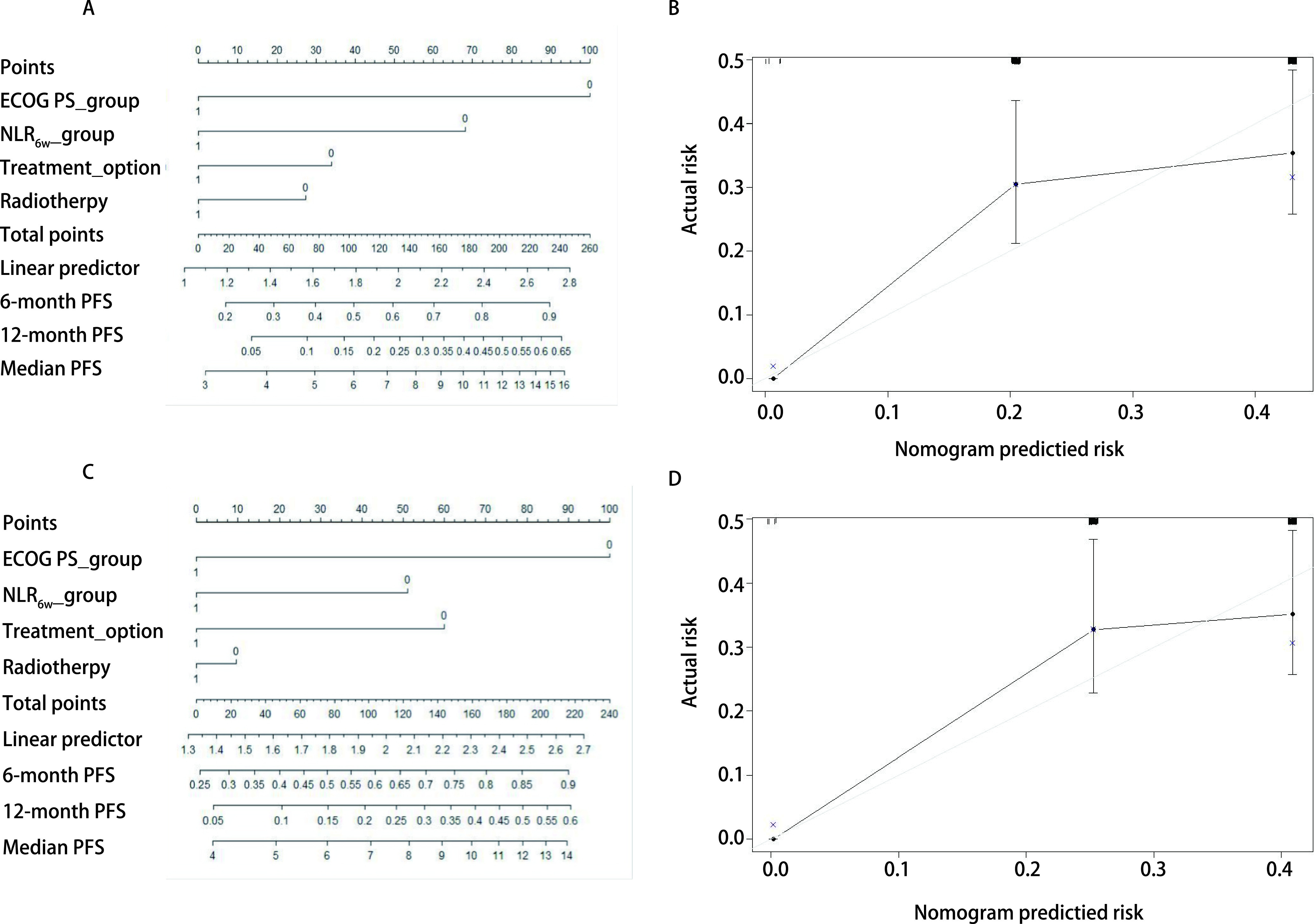
预测6个月及12个月无进展生存率的列线图及校准曲线图。A：ECOG评分和NLR_6w_的列线图；B：NLR_6w_预测6个月和12个月PFS概率的校准曲线；C：ECOG评分和NLR_12w_的列线图；D: NLR_12w_预测6个月和12个月PFS概率的校准曲线。 Nomogram predicting the probability of 6-months and 12-months progression-free survival (PFS) and calibration plots. A: A nomogram incorporating the presence of ECOG and NLR_6w_; B: Calibration curve for predicting the probability of 6-months and 12-months PFS using NLR_6w_; C: A nomogram incorporating the presence of ECOG and NLR_12w_; D: Calibration curve for predicting the probability of 6-months and 12-months PFS using NLR_12w_. ECOG PS_group: 0=PS 0-1, 1=PS 2; NLR_6w__group: 0=NLR_6w_ < 3, 1=NLR_6w_≥3; NLR_12w__group: 0=NLR_12w_ < 3, 1=NLR_12w_≥3; Treatment_option: 0=monotherapy, 1=combination therapy; Radiotherapy: 0=no radiotherapy, 1=receiving radiotherapy.

**图 4:**
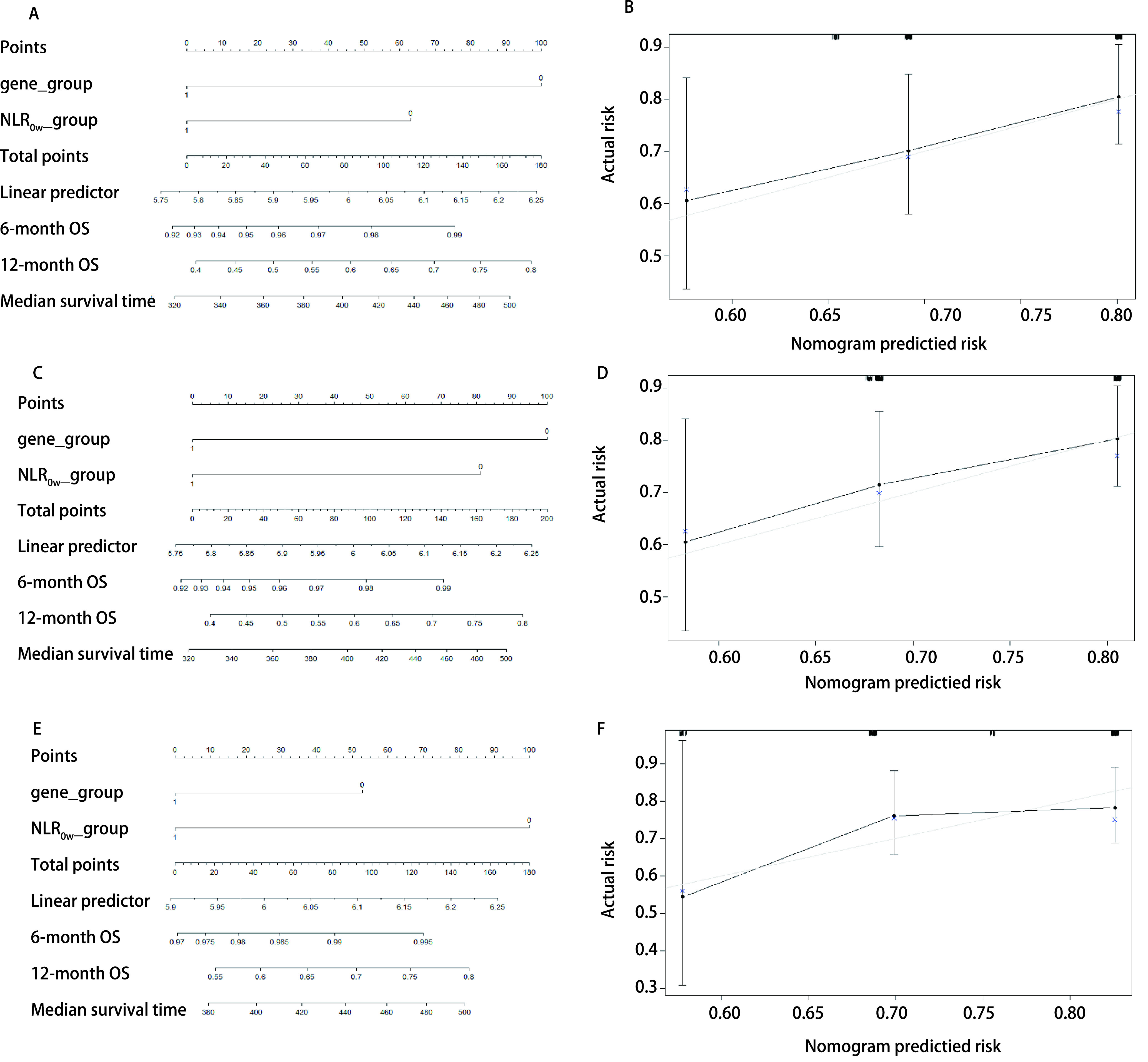
预测6个月及12个月总生存率的列线图及校准曲线图。A：基因突变类型和NLR_0w_的列线图；B：NLR_0w_预测6个月和12个月OS概率的校准曲线；C：基因突变类型和NLR_6w_的列线图；D：NLR_6w_预测6个月和12个月OS概率的校准曲线；E：基因突变类型和NLR_12w_的列线图；F：NLR_12w_预测6个月和12个月OS概率的校准曲线。 Nomogram predicting the probability of 6-months and 12-months OS and calibration plots. A: A nomogram incorporating the presence of gene mutation and NLR_6w_; B: Calibration curve for predicting the probability of 6-months and 12-months OS using NLR_6w_; C: A nomogram incorporating the presence of gene mutation and NLR_6w_; D: Calibration curve for predicting the probability of 6-months and 12-months OS using NLR_6w_; E: A nomogram incorporating the presence of gene mutation and NLR_12w_; F: Calibration curve for predicting the probability of 6-months and 12-months OS using NLR_12w_. Gene_group: 0=non-*EGFR* mutation, 1=*EGFR* mutation; NLR_0w__group: 0=NLR_0w_ < 3, 1=NLR_0w_≥3; NLR_6w__group: 0=NLR_6w_ < 3, 1=NLR_6w_≥3; NLR_12w__group: 0=NLR_12w_ < 3, 1=NLR_12w_≥3.

## 3 讨论

众所周知，对于晚期NSCLC患者，ICIs相对于传统化疗有更显著的疗效获益^[14-16]^，但是由于免疫治疗可能带来严重的不良反应，因此，预测使用ICIs的患者的疗效尤为重要。病理类型、PD-L1表达水平、TMB和肿瘤浸润淋巴细胞等可以有效地预测免疫治疗疗效，然而这些标记的检测方法较为复杂，并且只能反应某一个固定时间段的特征，无法实现动态预测，因此，通过常规实验室检查指标来预测免疫治疗疗效就显得尤为重要。在肿瘤发生发展的不同阶段，炎症反应发挥着不同的作用，并且其对肿瘤的免疫治疗反应有所影响，调节性T细胞（Tregs）具有免疫抑制功能，在肿瘤组织中聚集，在炎症反应中，这些细胞也被树突状细胞募集和激活^[17]^。骨髓来源的抑制性细胞（myeloid-derived suppressor cells, MDSCs）亦属于促炎细胞，它被炎症反应激活，并向特定部位聚集，促进血管形成，同时它也具有免疫抑制作用^[18]^。因此，肿瘤相关性炎症通过产生促炎和抗炎信号，导致肿瘤生长并逃避免疫监视。中性粒细胞、淋巴细胞等炎症标志物在此过程中发挥关键作用^[19]^。

在本回顾性研究中，我们评估了173例符合条件的NSCLC患者的临床特征和预后，并通过分析可能与NSCLC相关的外周血炎症相关指标（ANC、ALC、PLT、NLR、PLR等）对患者的预后进行预测。与既往部分研究不同的是，本研究根据不同时期的NLR、PLR数据构建了多个模型，从而可以动态地预测患者的疗效及生存。最终我们的数据显示NLR与患者预后密切相关，具体来讲，NLR_12w_ < 3的患者有更好的DCR，NLR_6w_和NLR_12w_ < 3的患者ORR更高并且有更长的PFS，在OS方面，不管何时，NLR < 3的患者的OS都更长。

既往诸多研究表明NLR、PLR等指标对NSCLC患者使用免疫治疗的预后有预测价值，且大部分研究表明基线的数值更有预测价值^[20-23]^。Capone等^[24]^的研究发现基线NLR≥5的接受免疫治疗的恶性黑色素瘤患者的OS和PFS显著低于NLR < 5的患者；Bartlett等^[25]^的研究也显示出基线NLR≥5与较短的OS相关（HR=2.0; 95%CI: 1.3-2.9）。而在本研究中，我们根据基线、治疗后6周以及12周三个时间段的NLR及PLR值预测患者的预后，然而我们并没有发现PLR在预测疗效及生存方面的价值，也没有发现基线NLR在患者免疫治疗疗效以及PFS方面的预测价值，只在预测OS方面发现基线NLR具有预测价值。可能的解释是，本研究纳入患者以复治患者居多，这类患者前期的化疗方案可能对免疫治疗前所测定的标志物的基线值有所影响。在PFS方面，我们发现接受过放疗的患者往往有更加良好的PFS获益，提示放疗在患者早期获益中的重要地位，但是对OS并没有影响，这可能是由于本研究样本量相对较少，有一定的局限性。需要注意的是，本研究中发现，接受ICIs单药治疗的患者有着更长的PFS，这可能是因为单药治疗的患者以一线初治患者为主，而这类患者往往有着更好的早期获益可能。在OS方面发现了基线NLR的预测价值可能是因为在NLR_0w_ < 3分组中，初治患者居多，这类患者免疫治疗进展后往往有更多后续治疗方案的选择，因此总生存期可以得到延长。为了评估本研究中的预测模型的准确性，我们还构建了列线图、计算一致性指数并绘制了校准曲线。可以发现本研究中的预测模型的一致性指数及校准曲线均显示出统计学意义。

本研究的不足之处主要是本研究为单中心研究，患者样本量有限，不同时期NLR的预后价值有待扩充病例数后进一步验证。此外，本研究的随访时间尚短，因此后续需要更长的随访时间来探索不同时期NLR对于生存预后的价值。最后，血液学指标的变化可能仅为化疗引起，需要只接受化疗的患者为对照组，然而，我们无法招募足够数量的患者，因为在目前的临床实践中单独化疗方案十分少见，后续将继续进行相关研究，建立验证队列。尽管有着如上局限性，但是免疫细胞在反应肿瘤免疫治疗时机体免疫炎症状态的作用仍不容忽视，这也是进行本研究的机制背景^[26-28]^。参照诸如NLR等简易实验室指标的动态变化进行免疫治疗方案的选择可能是未来的一个研究方向。

综上所述，不同时期的NLR可能是一种可以预测NSCLC患者免疫治疗疗效的临床生物标志物，并且其具有简易获取、动态监测的优势。未来可以通过更大样本量的研究进一步探索NLR等相关指标在NSCLC患者中的应用价值，为临床医生提供更方便有效的参考依据。

## References

[1] Sung H, Ferlay J, Siegel R (2021). Global cancer statistics 2020: GLOBOCAN estimates of incidence and mortality worldwide for 36 cancers in 185 countries. CA Cancer J Clin.

[2] Shaverdian N, Lisberg A, Bornazyan K (2017). Previous radiotherapy and the clinical activity and toxicity of pembrolizumab in the treatment of non-small-cell lung cancer: a secondary analysis of the KEYNOTE-001 phase 1 trial. Lancet Oncol.

[3] Alex F, Alfredo A (2020). Promising predictors of checkpoint inhibitor response in NSCLC. Expert Rev Anticancer Ther.

[4] Hanahan D, Weinberg R (2011). Hallmarks of cancer: the next generation. Cell.

[5] Banna G, Signorelli D, Metro G (2020). Neutrophil-to-lymphocyte ratio in combination with PD-L1 or lactate dehydrogenase as biomarkers for high PD-L1 non-small cell lung cancer treated with first-line pembrolizumab. Transl Lung Cancer Res.

[6] Zhang Z, Zhang F, Yuan F (2020). Pretreatment hemoglobin level as a predictor to evaluate the efficacy of immune checkpoint inhibitors in patients with advanced non-small cell lung cancer. Ther Adv Med Oncol.

[7] Petrova M, Donev I, Radanova M (2020). Sarcopenia and high NLR are associated with the development of hyperprogressive disease after second-line pembrolizumab in patients with non-small-cell lung cancer. Clin Exp Immunol.

[8] Simonaggio A, Elaidi R, Fournier L (2020). Variation in neutrophil to lymphocyte ratio (NLR) as predictor of outcomes in metastatic renal cell carcinoma (mRCC) and non-small cell lung cancer (mNSCLC) patients treated with nivolumab. Cancer Immunol Immunother.

[9] Diakos C, Charles K, McMillan D (2014). Cancer-related inflammation and treatment effectiveness. Lancet Oncol.

[10] Detterbeck F, Boffa D, Kim A (2017). The eighth edition lung cancer stage classification. Chest.

[11] Kim N, Yu J, Park H (2020). Incorporating sarcopenia and inflammation with radiation therapy in patients with hepatocellular carcinoma treated with nivolumab. Cancer Immunol Immunother.

[12] Motzer R, Ravaud A, Patard J (2018). Adjuvant sunitinib for high-risk renal cell carcinoma after nephrectomy: subgroup analyses and updated overall survival results. Eur Urol.

[13] Lorente D, Mateo J, Templeton A (2015). Baseline neutrophil-lymphocyte ratio (NLR) is associated with survival and response to treatment with second-line chemotherapy for advanced prostate cancer independent of baseline steroid use. Ann Oncol.

[14] Gandhi L, Rodriguez-Abreu D, Gadgeel S (2018). Pembrolizumab plus chemotherapy in metastatic non-small-cell lung cancer. N Engl J Med.

[15] Socinski M, Jotte R, Cappuzzo F (2018). Atezolizumab for first-line treatment of metastatic nonsquamous NSCLC. N Engl J Med.

[16] Langer C, Gadgeel S, Borghaei H (2016). Carboplatin and pemetrexed with or without pembrolizumab for advanced, non-squamous non-small-cell lung cancer: a randomised, phase 2 cohort of the open-label KEYNOTE-021 study. Lancet Oncol.

[17] Kim J, Kim B, Lee S (2020). Regulatory T cells in tumor microenvironment and approach for anticancer immunotherapy. Immune Netw.

[18] Giese M, Hind L, Huttenlocher A (2019). Neutrophil plasticity in the tumor microenvironment. Blood.

[19] Grivennikov S, Greten F, Karin M (2010). Immunity, inflammation, and cancer. Cell.

[20] Russo A, Russano M, Franchina T (2020). Neutrophil-to-lymphocyte ratio (NLR), platelet-to-lymphocyte ratio (PLR), and outcomes with nivolumab in pretreated non-small cell lung cancer (NSCLC): A Large Retrospective Multicenter Study. Adv Ther.

[21] Takada K, Takamori S, Yoneshima Y (2020). Serum markers associated with treatment response and survival in non-small cell lung cancer patients treated with anti-PD-1 therapy. Lung Cancer.

[22] Lalani A, Xie W, Martini D (2018). Change in neutrophil-to-lymphocyte ratio (NLR) in response to immune checkpoint blockade for metastatic renal cell carcinoma. J Immunother Cancer.

[23] Zer A, Sung M R, Walia P (2018). Correlation of neutrophil to lymphocyte ratio and absolute neutrophil count with outcomes with PD-1 axis inhibitors in patients with advanced non-small-cell lung cancer. Clin Lung Cancer.

[24] Capone M, Giannarelli D, Mallardo D (2018). Baseline neutrophil-to-lymphocyte ratio (NLR) and derived NLR could predict overall survival in patients with advanced melanoma treated with nivolumab. J Immunother Cancer.

[25] Bartlett E, Flynn J, Panageas K (2020). High neutrophil-to-lymphocyte ratio (NLR) is associated with treatment failure and death in patients who have melanoma treated with PD-1 inhibitor monotherapy. Cancer.

[26] Kargl J, Busch S, Yang G (2017). Neutrophils dominate the immune cell composition in non-small cell lung cancer. Nat Commun.

[27] Gregory A, Houghton A (2011). Tumor-associated neutrophils: new targets for cancer therapy. Cancer Res.

[28] Fridlender Z, Sun J, Kim S (2009). Polarization of tumor-associated neutrophil phenotype by TGF-beta: "N1" versus "N2" TAN. Cancer Cell.

